# Significant increase in quantity and quality of knee arthroplasty related research in KSSTA over the past 15 years

**DOI:** 10.1007/s00167-021-06555-2

**Published:** 2021-04-10

**Authors:** Stephanie Kirschbaum, Thilo Kakzhad, Fabian Granrath, Andrzej Jasina, Jakub Oronowicz, Carsten Perka, Sebastian Kopf, Clemens Gwinner, Matthias Pumberger

**Affiliations:** 1 Center for Musculoskeletal Surgery, Charité, University Medicine Berlin, Charitéplatz 1, 10117 Berlin, Germany; 2 Center of Orthopaedics and Traumatology, Brandenburg Medical School Theodor Fontane, Brandenburg an der Havel, Germany

**Keywords:** Total knee replacement, Total knee arthroplasty, Authorship characteristics, Publishing characteristics, Trends in research

## Abstract

**Purpose:**

This study aimed to evaluate both publication and authorship characteristics in Knee Surgery, Sports Traumatology, Arthroscopy journal (KSSTA) regarding knee arthroplasty over the past 15 years.

**Methods:**

PubMed was searched for articles published in KSSTA between January 1, 2006, and December 31st, 2020, utilising the search term ‘knee arthroplasty’. 1288 articles met the inclusion criteria. The articles were evaluated using the following criteria: type of article, type of study, main topic and special topic, use of patient-reported outcome scores, number of references and citations, level of evidence (LOE), number of authors, gender of the first author and continent of origin. Three time intervals were compared: 2006–2010, 2011–2015 and 2016–2020.

**Results:**

Between 2016 and 2020, publications peaked at 670 articles (52%) compared with 465 (36%) published between 2011 and 2016 and 153 articles (12%) between 2006 and 2010. While percentage of reviews (2006–2010: 0% vs. 2011–2015: 5% vs. 2016–2020: 5%) and meta-analyses (1% vs. 6% vs. 5%) increased, fewer case reports were published (13% vs. 3% vs. 1%) (*p* < 0.001). Interest in navigation and computer-assisted surgery decreased, whereas interest in perioperative management, robotic and individualized surgery increased over time (*p* < 0.001). There was an increasing number of references [26 (2–73) vs. 30 (2–158) vs. 31 (1–143), *p* < 0.001] while number of citations decreased [30 (0–188) vs. 22 (0–264) vs. 6 (0–106), *p* < 0.001]. LOE showed no significant changes (*p* = 0.439). The number of authors increased between each time interval (*p* < 0.001), while the percentage of female authors was comparable between first and last interval (*p* = 0.252). Europe published significantly fewer articles over time (56% vs. 47% vs. 52%), whereas the number of articles from Asia increased (35% vs. 45% vs. 37%, *p* = 0.005).

**Conclusion:**

Increasing interest in the field of knee arthroplasty-related surgery arose within the last 15 years in KSSTA. The investigated topics showed a significant trend towards the latest techniques at each time interval. With rising number of authors, the part of female first authors also increased—but not significantly. Furthermore, publishing characteristics showed an increasing number of publications from Asia and a slightly decreasing number in Europe.

**Level of evidence:**

IV.

## Introduction

Although knee arthroplasty is a successful procedure to treat end-stage osteoarthritis, 20% of patients are not satisfied with their outcome [15]. Therefore, notable variations and developments in surgical techniques with many controversies arose over time [22]. Especially as the number of knee arthroplasties is continuously rising worldwide, high-quality research and evidence-based recommendations are required to evaluate those techniques and improve outcome. Following, there is increasing interest in publication quality and internationalisation as well as gender disparity concerning authorship in the orthopaedic literature [3, 4, 7, 12]. However, few literature sources have evaluated the level of evidence (LOE) in the field of knee arthroplasty [8]. Even less studies evaluate shift of trends in research topics but only represent felt trends or experts’ agreements [1, 22]. Understanding changes in publishing characteristics over time, by topic, by origin and by gender are critical, especially with the rising demands of publishing in academia. Therefore, the aim of this study was to evaluate shift of trends in article design and research topic identifying topics which have been excessively investigated, those who did get less attention by now or those who are starting to become popular. The authors hypothesised that there was a significant shift regarding the research topics within the last 15 years concerning the field of knee arthroplasty. This analysis might help researchers to choose the focus of future research topics. Furthermore, evaluation of the level of evidence as well as trends in study designs and publication characteristics shows where the quality of research stands now and how valuable studies should be designed. The authors assumed that there is a significant increase regarding the LOE and number of cases included in research articles over time. Some authors described an increasing part of female first authorships in orthopaedic research articles [19]. However, as most of the experts gaining national and international visibility in the field of knee arthroplasty are male not representing the described trend, the recent study also evaluated trends in authorship characteristics. The authors hypothesised that this trend might fit general orthopaedic research but not represents the state of knee arthroplasty related research.

**Fig. 1:**
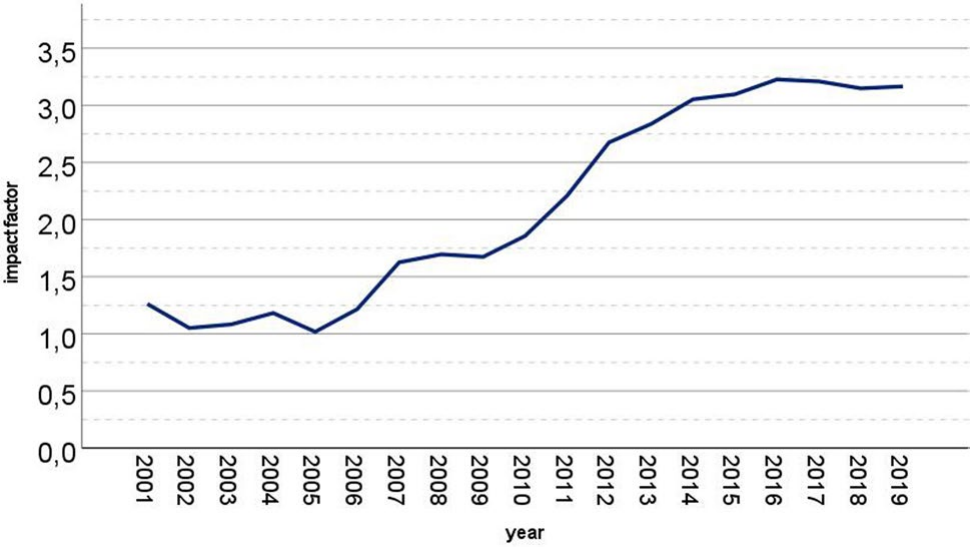
Development of the two-year impact factor of KSSTA journal from 2001 to 2019

Knee surgery, sports traumatology, arthroscopy (KSSTA) journal represents an orthopaedic high-impact journal which requires high research standards for publication achieving an impact factor of 3.166 in 2019 (Fig. 1). While the initial focus was innovative sports traumatology as well as reconstructive knee surgery, KSSTA became increasingly interesting for articles concerning knee arthroplasty to cover all aspects of knee-related surgery over the last 15 years. Because of this innovative perspective and broad spectrum, KSSTA was selected for the analysis described.

## Methods

A literature search was performed on 04th March 2021 in PubMed for articles published (online or in print) in KSSTA journal between January 1, 2006, and December 31, 2020. The following search terms were used:

“Knee surgery, sports traumatology, arthroscopy: official journal of the ESSKA”[Journal] AND ((“2006/01/01”[Date—Publication]: “2020/12/31”[Date—Publication])) AND (knee arthroplasty).

**Fig. 2:**
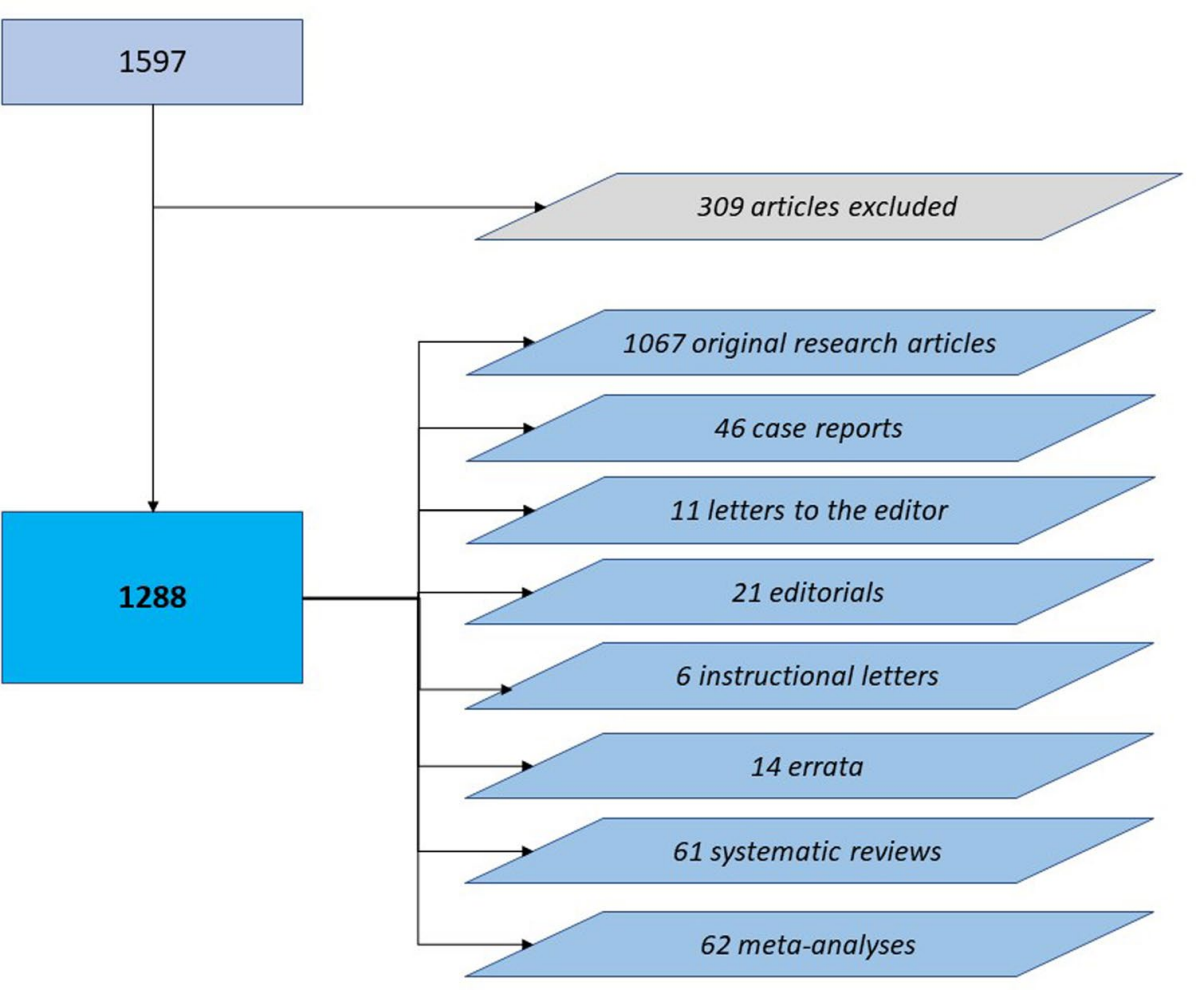
Flowchart of article enrolment

In total, 1597 articles were identified, representing 25% of all 6289 published articles in KSSTA within the chosen time interval (((“Knee surgery, sports traumatology, arthroscopy: official journal of the ESSKA”[Journal]) AND ((“2006/01/01”[Date—Publication]: “2020/12/31”[Date—Publication])))). Articles not related to the topic of knee arthroplasty (*n* = 309) were excluded. An overview of the remaining 1288 articles (original research article, case reports, letters to the editor, editorials, instructional letters, errata, systematic reviews, meta-analyses) is shown in Fig. 2. Articles were subdivided into three time intervals: (1) January 1, 2006 to December 31, 2010; (2) January 1, 2011 to December 31, 2015; and (3) January 1, 2016 to December 31, 2020.

To better assess present and past trends in publishing, the type of study (clinical therapeutic, clinical diagnostic, clinical prognostic, clinical economic, anatomic study, biomechanical/kinematic evaluation, basic science and other) was recorded [2]. Furthermore, the main topics (unicondylar knee arthroplasty (UKA), total knee arthroplasty (TKA), TKA after osteotomy, revision-UKA to TKA, revision-TKA to TKA, patellofemoral joint arthroplasty (PFJ) and special topics (robotics, alignment/implant positioning, navigation and computer-assisted surgery, implant/insert design, rehabilitation and general outcome, perioperative management, patella issue, patient-specific instrumentation and individual implant, infection and other complications, COVID-19 and others) were independently evaluated by two reviewers.

Continent of origin, number of authors and gender of the first author were analysed. A Google search of all first authors was performed to identify their gender. If proper author identification was not possible, the Google database was used to analyse common name patterns. If the author’s gender could not be identified with either method, the gender was marked as “unknown”. Authorship characteristics were, additionally to interval analysis, evaluated by year to detect even minimal changes in publishing characteristics especially regarding the number and gender distribution of authorship.

The number of used references, number of times an article was cited (= citations) in Google Scholar search. The level of evidence (LOE) reported by KSSTA was documented as well. Additionally, the number of cases and study design (prospective, retrospective) were documented for every original research article.

If reported, the type of patient-reported outcome measurement (PROM) was documented, namely the Knee Society Score (KSS), (Oxford Knee Score (OKS), Western Ontario and McMaster Universities Osteoarthritis Index (WOMAC), Short Form-12 (SF-12), Short Form-36 (SF-36), EuroQol 5D (EQ-5D), Visual Analogue Scale (VAS), Hospital for Special Surgery Score (HSS), American Knee Society Score (AKSS), Knee Injury and Osteoarthritis Outcome Score (KOOS), Forgotten joint Score (FJS), University of California at Los Angeles (UCLA), Knee Society Knee Score, Knee Society Function Score (KSKS & KSFS) and others.

### Statistical analysis

SPSS Version 26 (IBM®) was used for statistical analysis. Comparison of the publication characteristics of nominal variables was performed using chi-squared or Fisher’s test. The Kruskal–Wallis test was used for comparisons of publication characteristics over time involving count variables. The data distribution of each metric parameter was checked using the Kolmogorov–Smirnov test. If data showed no normal distribution, statistical evaluation was expressed as medians [with ranges]. If the data showed a normal distribution, the results were presented as means $\pm$ standard deviation. The Mann–Whitney test was used to compare the subgroup analysis of metric data because the distribution was not normal. *p* < 0.05 was considered significant.

## Results

KSSTA showed an increasing number of publications concerning knee arthroplasty-associated themes over the last 15 years (Table 1).

**Table 1:** Publishing characteristics regarding the total number of publications, references and citations as well as continent of origin over time

	2006–2010	2011–2015	2016–2020	
Total number of articles published	153	465	670	
References used per article	26 [2–73]	30 [2–158]	31 [1–143]	*p* < 0.001*
Number of times an article was cited	30 [0–188]	22 [0–264]	6 [0–106]	*p* < 0.001*

Continent		*n*		*n*		*n*
Europe	56.2%	86	46.9%	218	51.8%	347
Asia	35.3%	54	45.4%	211	36.7%	246
North America	3.9%	6	4.9%	23	9%	60
Others	4.6%	7	2.8%	13	2.5%	37

*Significant results

### Type of article and study, main and special topics over time

Most of the articles published within the last 15 years were original articles (*n* = 1066, 82.8%), followed by meta-analyses (*n* = 63, 4.9%), systematic reviews (*n* = 6, 4.7%), and case reports (*n* = 46, 3.6%). Editorials made up 1.6% (*n* = 21), errata 1.1% (*n* = 14), letters to the editor 0.9% (*n* = 11) and instructional letters 0.5% (*n* = 6) of all knee arthroplasty related publications between 2006 and 2020.

**Fig. 3:**
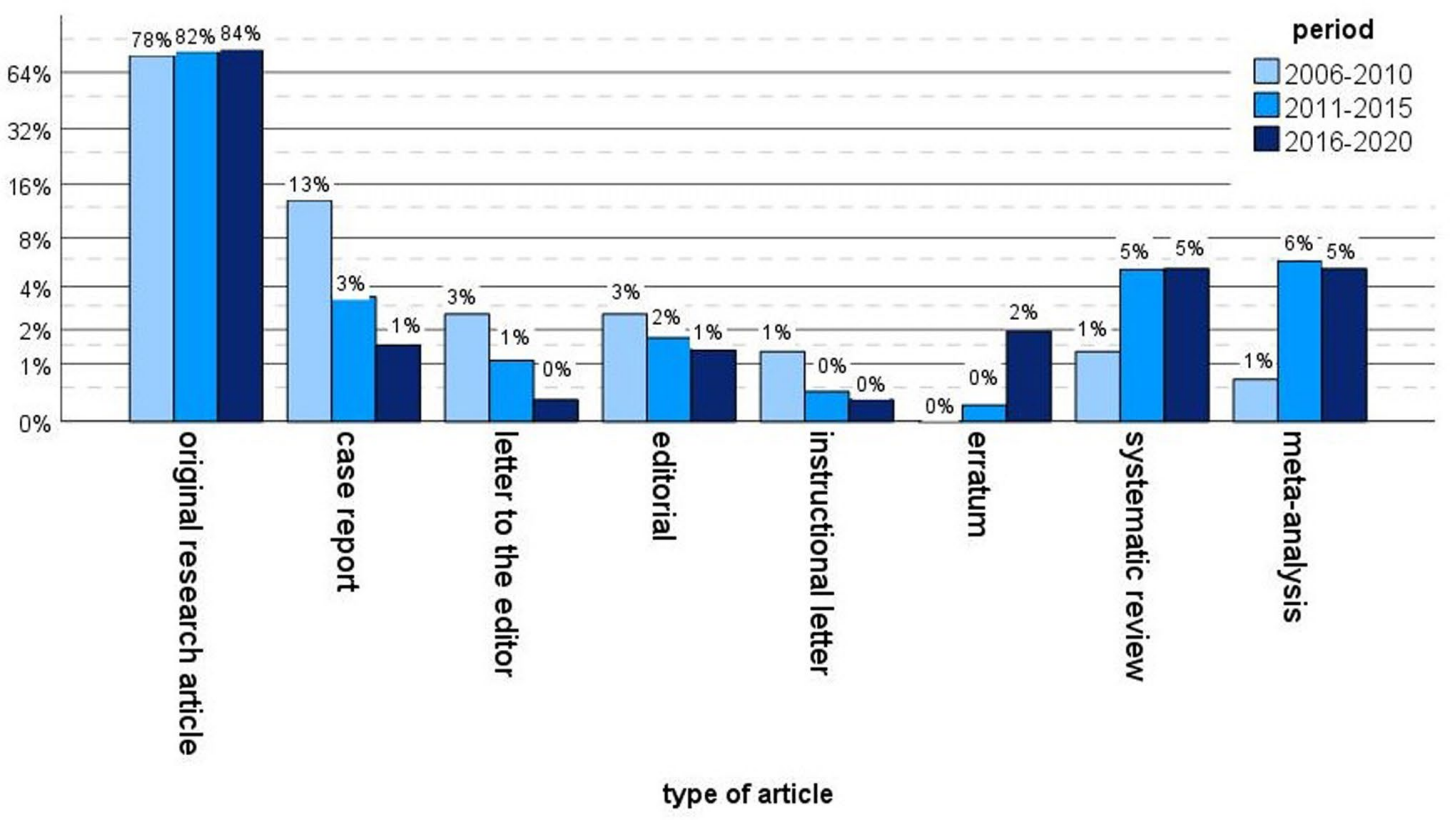
Comparison of the types of articles published within the three time intervals. Logarithmic scale

Comparison of the publishing trends within the three time intervals revealed an increase in the number of reviews (1.3% vs. 5.2%) and meta-analyses (0.7% vs. 5.2%) while fewer case reports (13.1% vs. 1.5%) were published (*p* < 0.001, Fig. 3).

**Fig. 4:**
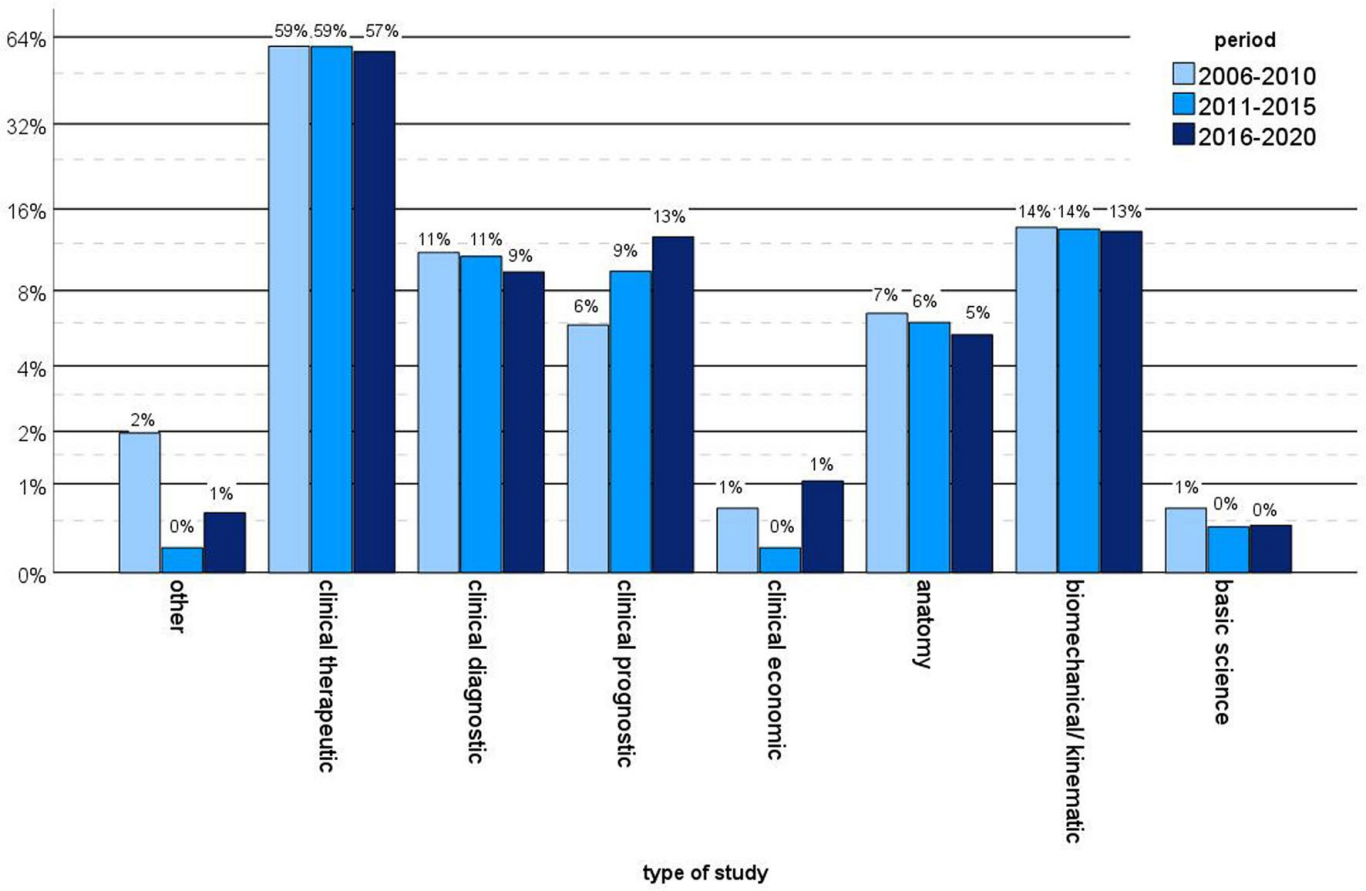
Comparison of study types over the years. Logarithmic scale

Most of the published studies were clinical therapeutic studies (58.2%, *n* = 749), followed by biomechanical/kinematic studies (13.4%; *n* = 173), clinical prognostic studies (10.7%; *n* = 138) and clinical diagnostic studies (10.1%; *n* = 130). Anatomical studies represented 5.7% (*n* = 74), clinical economic studies 0–7% (*n* = 9) and 0.5% were basic sciences studies (*n* = 6). Nine studies did not meet any of this topic (0.7%). Whereas the percentage of clinical therapeutic studies slightly decreased from 59.5% (*n* = 91) between 2006 and 2010 to 57% (*n* = 382) between 2016 and 2020, the raw numbers of published clinical therapeutic articles on knee arthroplasty increased tremendously. There was an increase in clinical prognostic studies from 5.9% (*n* = 9) to 12.7 (*n* = 85) whereas clinical diagnostic studies (11.9% vs. 9.4%, *n* = 17 vs. 63) and biomechanical/kinematic studies (13.7% vs. 13.3%, *n* = 21 vs. 89) showed no relevant changes over the three time intervals (*p* = 0.305, Fig. 4).

Most studies evaluated the field of TKA (74.4%; *n* = 958), followed by UKA (12%; *n* = 152) and patellofemoral joint replacement (PFJ; 1.6%; *n* = 21). Overall, 5.6% of the published studies in the KSSTA journal focused on revision surgery in knee arthroplasty. Comparison of the three time intervals revealed that the topic of TKA was covered in 78.4% (*n* = 120) of the articles from 2006 to 2010, 77.8% (*n* = 362) between 2011 and 2015, and 71% (*n* = 476) between 2016 and 2020. Regarding UKA, 11.1% (*n* = 17) of all the articles published between 2006 and 2010 treated this topic, 11.4% (*n* = 53) between 2011 and 2015, and 12.4% (*n* = 83) between 2016 and 2020. These changes did not reach statistical significance (*p* = 0.187).

**Fig. 5:**
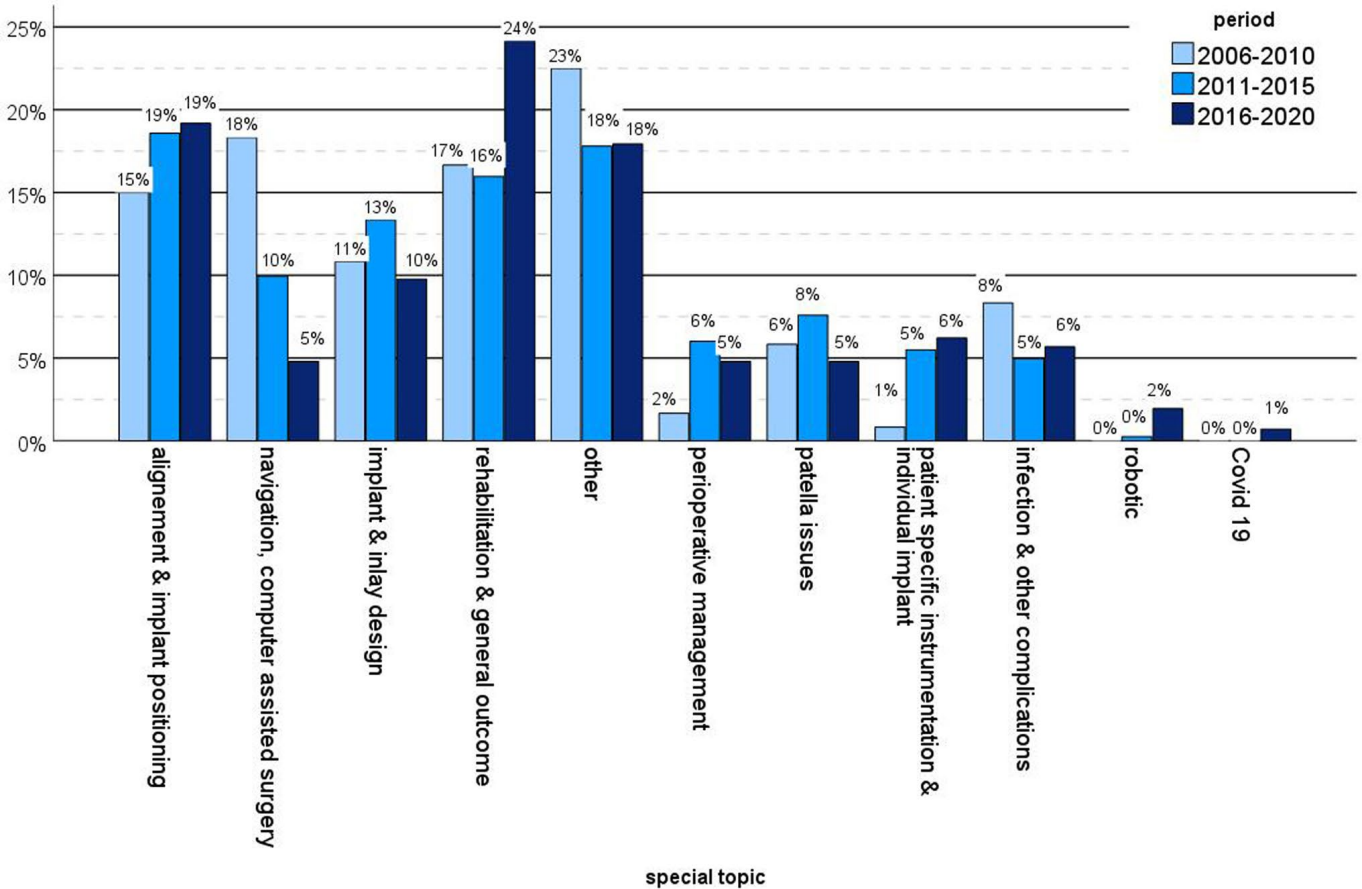
Change in the topics of research interest between each time period

Figure 5 demonstrates the shift of research interest within the described special topics over time. From 2006 to 2010, 16.3% (*n* = 25) of the articles researched navigation and computer-assisted surgery, and 4.9% (*n* = 33) focused on that topic from 2016 to 2020. Additionally, interest in perioperative management increased from 0.2% (*n* = 3) to 4.9% (*n* = 33), interest in rehabilitation and general outcome increased from 13.1% (*n* = 20) to 24.7% (*n* = 165) and interest in patient-specific instrumentation and implants increased from 0.7% (*n* = 1) to 6.3% (*n* = 42) between 2006 until 2010 and 2016 until 2020 (*p* < 0.001). Covid-19 associated research reached 1% (*n* = 5) of all publication within the latest interval of 2016 until 2020. The topic of robotic-assisted surgery also just arose within the last interval between 2016 and 2020 (2.2%, *n* = 15).

### Origin of article

Most of the publications were from Europe (50.5% (*n* = 651), followed by Asia with 39.7% (*n* = 511) and North America 6.9% (*n* = 89). The number of publications from Europe decreased from 56.2% (*n* = 86) of all publications in 2006–2010 to 51.8% (*n* = 347) in 2016–2020 (Table 1). Additionally, the number of publications from Asia increased from 35.3% (*n* = 54) to 45.4% (*n* = 211) between 2011 and 2015 and again returned to 36.7% (*n* = 246) between 2016 and 2020. North America increased from 3.9% (*n* = 6) of all publications between 2006 and 2010 to 9% (*n* = 60) between 2016 and 2020. Those changes were significant (*p* = 0.005). Table 1 provides a detailed overview.

### Authorship and gender characteristics

**Fig. 6:**
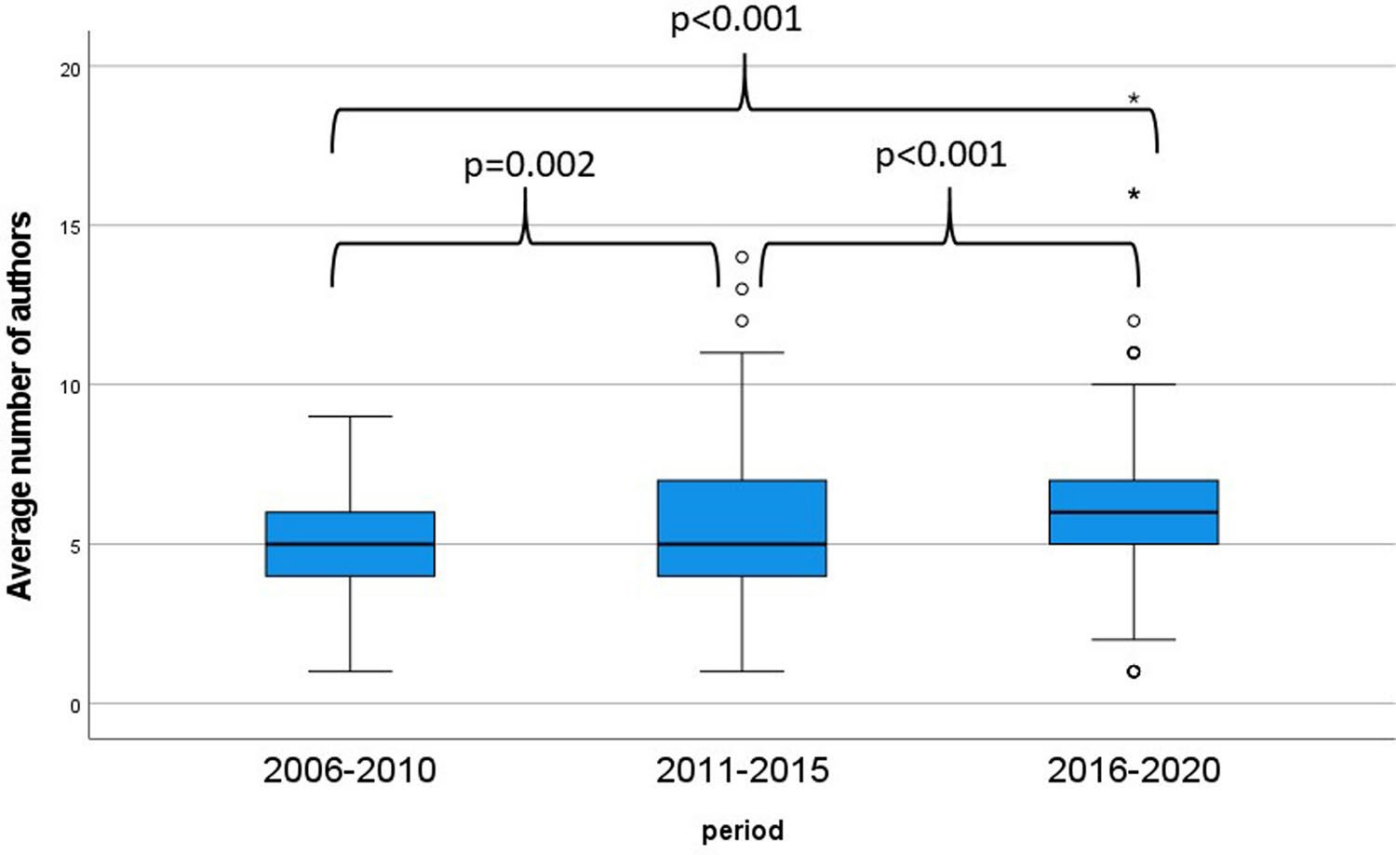
Average number of authors per article between each time period

The average number of authors was 5 [1–9] between 2006 and 2010, 5 [1–14] between 2011 and 2015 and 6 [1–19] between 2016 and 2020 (*p* < 0.001). This significant rise is demonstrated in detail by Fig. 6.

**Fig. 7:**
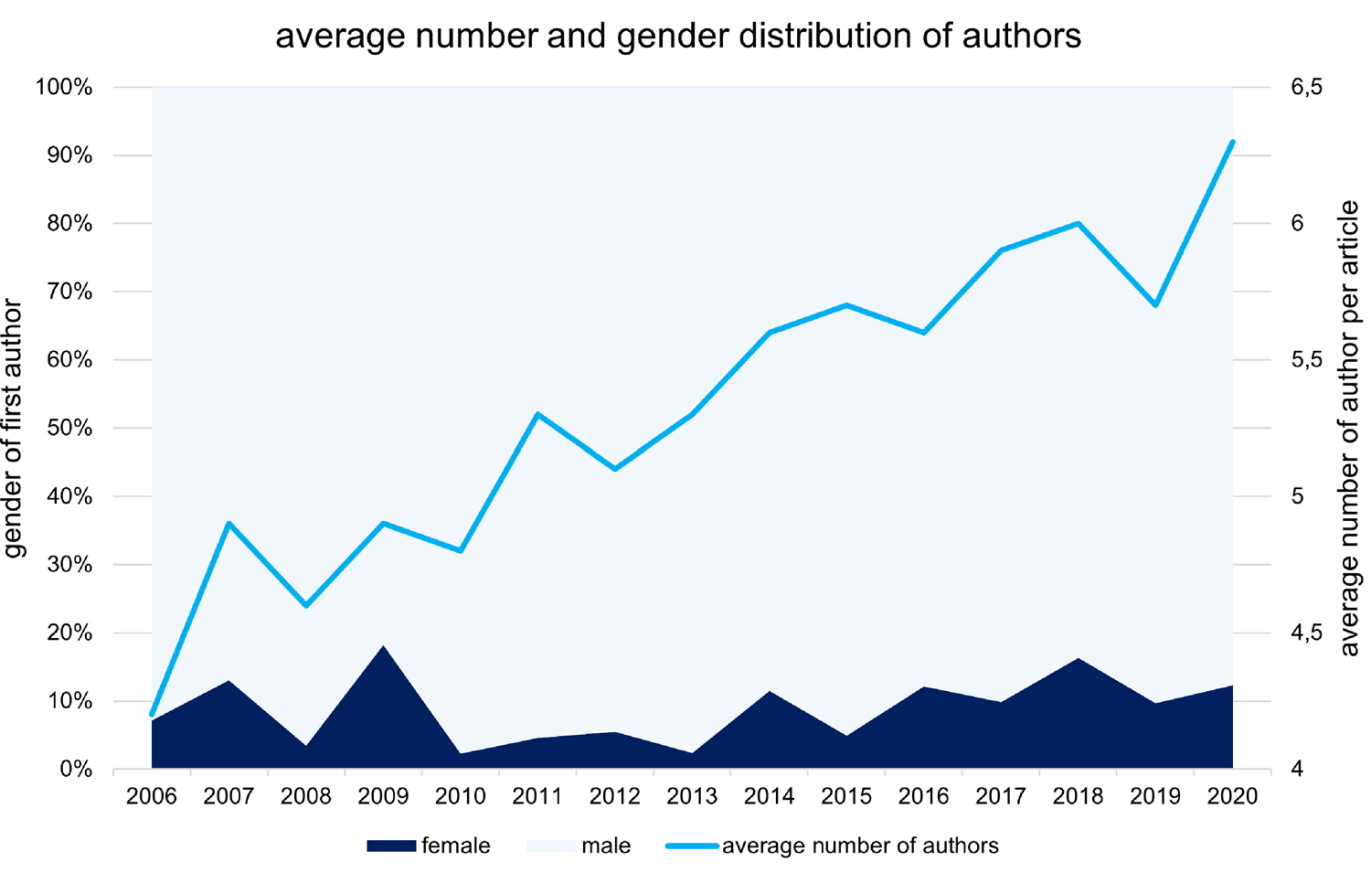
Development of average number and gender distribution of authors per publication over time

In 85 cases (6.6%), gender identification of the first author was not possible. Most (90.5%; *n* = 1090) of the remaining authors publishing in KSSTA journal about knee arthroplasty within the last 15 years were male. The percentage of female authors decreased between 2006 and 2010 (8.4%; *n* = 12) and between 2011 and 2015 (6.5%; *n* = 28) and increased significantly between 2016 and 2020 (11.7%; *n* = 74, *p* = 0.005, Fig. 7). Comparison of female authorship proportion between the initial interval of 2006–2010 and the latest interval 2016–2020 showed no significance (*p* = 0.252).

### Number of references, citations, level of evidence (LOE), number of analysed cases and study design

A significant increase was observed in the average number of references used between 2006 and 2015 (*p* < 0.001) whereas no significant change in the number of references was seen between 2011 and 2020 (*p* = 0.192). Number of times an article was cited significantly decreased between each time interval (each *p* < 0.001, Table 1).

Because the LOE was not reported regularly from 2006 to 2010 (*n* = 3), only a comparison between the time ranges of 2011–2015 [2.7 ± 1.1 (1–5)] and 2016–2020 [2.8 ± 1 (1–5)] was performed. The LOE showed no significant change over time (*p* = 0.439).

Evaluating the number of cases examined in the original articles, a significant increase was observed over all intervals (*p* = 0.001). Between 2006 and 2010 average of 115 [5–1474] cases were included. 2011 and 2015 193 cases [6–14785] and 2016 and 2020 1565 cases [1–290601].

A significant increase was found in the percentage of retrospective study designs between 2006–2011 and 2016–2020 (29.8% (*n* = 34) vs. 43.6% (*n* = 222) whereas percentage of prospective study design decreased meanwhile from 67.5% (*n* = 77) to 51.3% (*n* = 261, *p* < 0.001). Cadaver studies constantly increased from 2.6% (*n* = 3) between 2006 and 2010 to 5.1% (*n* = 26).

### Patient-reported outcome measurement (PROM)

**Fig. 8:**
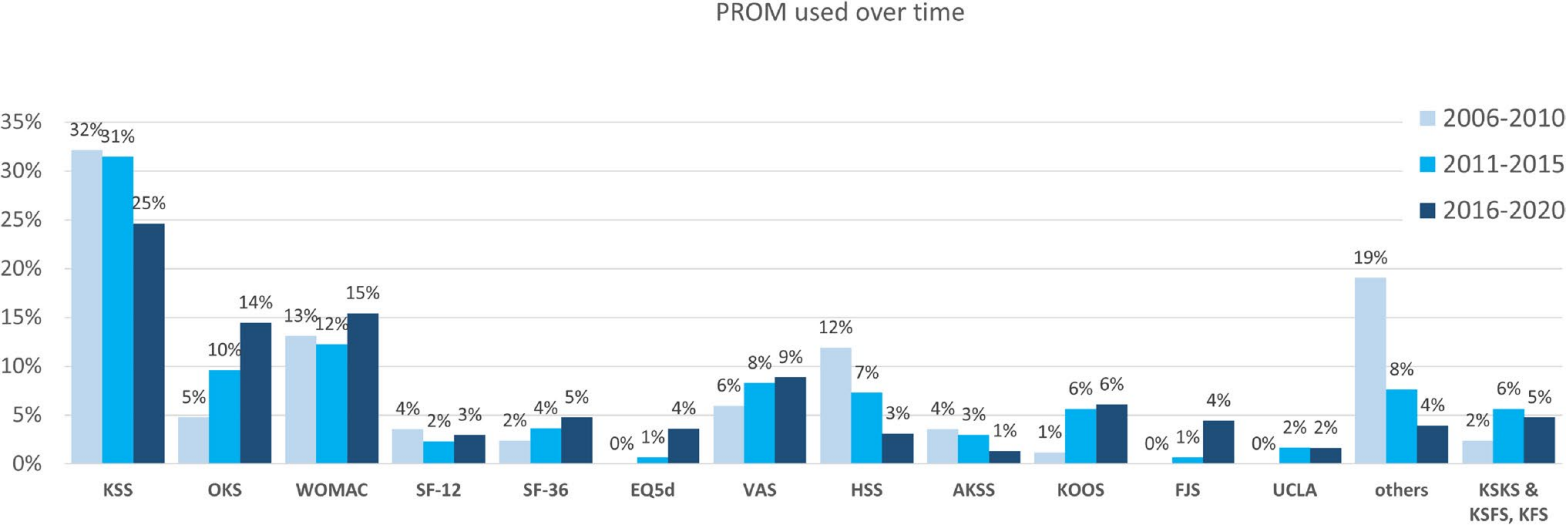
Detailed comparison of the PROMS used within the chosen time intervals. *KSS* knee society score, *OKS* oxford knee score, *WOMAC* Western Ontario and McMaster Universities Osteoarthritis Index, *SF-12* short form-12, *SF-36* short form-36, *EQ-5D* EuroQol 5D, *VAS* visual analogue scale, *HSS* Hospital for Special Surgery Score, *AKSS* American Knee Society Score, *KOOS* Knee Injury and Osteoarthritis Outcome Score, *FJS* Forgotten Joint Score, *UCLA* University of California at Los Angeles, *KSKS* Knee Society Knee Score, *KSFS* Knee Society Function Score, *PROM* patient-reported outcome measurement

Overall, 40.9% (*n* = 527) of the publications reported the use of PROM. The overall use of PROM increased over time (2006–2010: 35.9% (*n* = 55); 2011–2015: 37.2% (*n* = 173); 2016–2020: 44.6% (*n* = 299); *p* = 0.018). In total, the KSS was the most used PROM (27.1%; *n* = 772), followed by the WOMAC score (14.3%; *n* = 142) and OKS (12.2%; *n* = 121). While the use of the KSS (32–25%) and HSS (12–3%) decreased over the three time intervals, the use of the OKS (5–14%), WOMAC score (13–15%), KSKS & KSFS (2–5%) and KOOS (1–6%) increased (Fig. 8).

## Discussion

This study aimed to better understand the publishing characteristics and trends in knee arthroplasty-related articles within the last 15 years. Overall, the number of arthroplasty-associated publications has increased significantly over the last 15 years, demonstrating a decreasing interest in case reports, while systematic reviews and meta-analyses have become popular. Interest in original articles, particularly concerning clinical therapeutic studies, was stable, while interest in prognostic and kinematic studies increased. Furthermore, special topics focused always on the latest techniques during each interval so that interest in navigation vanished while interest in patient-individualised surgery or robotics was enhanced. In particular, Asian countries, as well as countries in North America, showed an increase in submissions during the last 15 years while bisecting the percentage change to European submissions.

Between 2016 and 2020, nearly four times as many arthroplasty-associated articles were published in KSSTA than between 2006 and 2010. This extraordinary increase might be due to the changed focus of KSSTA adding arthroplasty-associated topics to journal research interests. An additional reason might be the changed submission options, particularly regarding open access possibility (since 2007), offering researchers more academic visibility. Furthermore, publication in KSSTA became more attractive over time regarding the increasing impact factor within the last 15 years (2005—IF 1.605; 2015—3.444 IF; 2019—3.518 IF), resulting in a higher prestige of published articles and better academic visibility.

Although case reports have become rare within the last 15 years, the publication of systematic reviews as well as meta-analyses has increased considerably. This trend was not only observed in KSSTA but also was already described in an editorial in *The British Medical Journal* in 2015 [17]. The number of case reports declined from 149 in 1990 to 37 in 2005, while more original studies were published. Another study evaluated the publishing options in the top 25 medical journals and stated that 32% of journals did not publish case reports, and another 36% published them in some modified format (e.g., online only or two issues per year) [6]. These findings mirror the recent European development that case reports have come under disfavour in the medical scientific community and are often disparaged to the lowest rung of the hierarchy of study design [5]. Furthermore, they are commonly not considered relevant for doctoral theses or desired for the Doctor of Philosophy (Ph.D.) degree; therefore, they are not considered valuable. Instead, interest in systematic reviews as well as meta-analyses have risen, reflecting their increasing relevance and value in the current climate of evidence-based medicine.

Among the original articles, a notable shift concerning the special topics of published articles was observed. Although interest in patient-specific implants and instrumentation, TKA positioning and alignment have increased constantly, and research in the field of robotics has only emerged within the last 5 years, interest in navigation, as well as computer-assisted surgery, has nearly vanished. Due to the outstanding pandemic situation beginning at the end of 2019 the topic of COVID-19 just arose within the last year. Although some foci have changed throughout the years, research issues concerning the TKA outcome and improving rehabilitation have remained important topics throughout all the time intervals. This observation is critical because a proportion of patients (up to 20%) are unsatisfied with their outcome after TKA [15]. Therefore, new strategies, implants, surgical techniques and rehabilitation programmes are evaluated in KSSTA following current trends to achieve optimal surgical outcomes.

The percentage of European publications has shown a continuous decrease over the last 15 years, while that of North America demonstrated a major increase. Although Asian publication increased between 2006 and 2015 the recent study revealed a major decrease within the last interval. This drop might be caused by a pandemic situation starting in Asia in 2019 and might have influenced Asian countries earlier than European or American countries also in research submission. Bradley et al. described, for example, a significant increase in publications from Asia in *The Bone and Joint Journal* between 2004 and 2018 (7.9–16.7%). The increasing number, particularly of Asian publications, was also described by other authors [2, 9, 14]. Zhi et al. evaluated 143,138 orthopaedic articles published from 2005 to 2014 concerning the LOE and country of origin. Although the United States led the field regarding the quality and quantity of orthopaedic research, they also found that China demonstrated considerable progress in orthopaedic research, not only in quantity but also in quality [23]. The great interest in orthopaedic research, particularly regarding Chinese publication behaviour over the last 15 years, might be due to musculoskeletal disorders becoming a major public health problem, as reported in *Lancet* 2017 [24]. To improve quality and quantity as well as to gain international visibility in the field of orthopaedics, the Department of Health Sciences of the National Natural Science Foundation of China invested approximately 145 million € in funding orthopaedic research within the last 10 years [16]. However, the reason might also be due to the increased international visibility of KSSTA within recent years.

Increases in the number of authors per publication have been found in multiple fields of medicine. A previous study evaluated publication characteristics in the American *The Journal of Bone and Joint Surgery* (JBJS) and confirmed a significant increase in authorships per orthopaedic publication over time [4]. Multiple reasons are suggested. First, the increase in the sophistication of research questions and the complexity of methods requires a well-equipped research team and a greater number of team members. Previous studies have already found an increasing percentage of M.D.s and Ph.D.s being last authors over a 15-year time interval [2, 9]. Hanzlik et al. also described an increasing level of evidence in JBJS over the last 30 years, likely supporting the hypothesis that a larger team is necessary for sophisticated and complex research issues [10]. Another reason might be an increasing acknowledgement of colleagues and research assistants who are currently given credit for their contribution, leading to an increased number of authors. Recent studies have found an increasing proportion of authors who are non-clinician scientists or others (i.e., authors with neither an M.D. degree nor an advanced research degree) over time, confirming this hypothesis [4, 14, 18]. Furthermore, academic visibility and, therefore, authorship have become more important for national and international prestige, resulting in increased patient acquisition. During the increasing economisation of medicine, academic visibility is also an essential economic factor because gaining funds guarantee the financing of projects, equipment and often co-workers. Both reasons result in the pressure to publish and result in phrases such as ‘publish or perish’.

Throughout the whole period, most of the articles were published by male first authors. Although the absolute number of first female authors increased over time, no significant increase was observed in the percentage of women gaining visibility as a first author in the field of knee arthroplasty between first and latest interval. The positive trend reported by other authors, evaluating publishing characteristics in the topic of foot and ankle as well as hand surgery, was not confirmed by the recent study [2, 9]. In contrast, Brown et al. failed to find a match between the growth of practising female orthopaedic surgeons and an increase in senior authorships by women over the last 30 years [3]. Considering the aforementioned studies, an increase in female authorship is more likely to be observed in journals treating small joints but not larger joints such as the knee and hip. Hiller et al. evaluated women’s authorships among *JBJS*, *The American Journal of Sports Medicine* and *Clinical Orthopaedics and Related Research* between 2006 and 2017 [11]. They found a modest increase in general female first authorship (11% in 2006 and 17% in 2017) but no increase in female last authorship (9% in 2006 and 10% in 2017). The authors also demonstrated that the percentage of female authorships within orthopaedic research strongly depends on subspecialty.

An increasing quality of published papers in KSSTA can be assumed due to the increasing number of cases evaluated in original articles. This development is probably also impacted by the increasing number of register studies which are published over the last years [21]. The continuous drop of average citations by each period can be explained by the young age and therefore less time for being citated as well as the rising number of overall publications. Unlike recent studies [1, 8], the current analysis failed to find an increasing LOE over time, likely because the LOE was not regularly reported between 2006 and 2010. The mentioned articles demonstrated an increase in the LOE of publications between 2004 and 2018, confirming the increasing quality and perhaps the sophistication of research questions over time to confirm the mentioned hypothesis. The observed increase in the number of references per article might be explained by the increasing number of orthopaedic research articles in general or an improved technical possibility of providing easier access to the literature [13].

To assess postoperative outcomes, the use of PROM has become an essential part of prospective and retrospective study designs. Because many established PROMs, such as KSS or HSS, show ceiling effects after arthroplasty and are not suitable to access an increasing number of active and demanding patients, a shift occurs towards more modern PROMS, such as KOOS or FJS, which provide higher responsiveness and lower ceiling effects than traditional PROMs [20].

The current study has several limitations. First, some studies related to the topic of knee arthroplasty might were sorted out by the filtering process because they did not contain the keyword or any related description in either title, keywords nor abstract. Second limitation represents the classification procedure concerning the main and special topic of publications. In order to decrease selection bias, every publication was classified by two independent reviewers and in case of any differences, the classification was set in a consensus approach. Another limitation represents the simplified origin of publication to the continent instead of country. As KSSTA reaches out to a lot of different countries resulting in a large number of data authors decided to simplify publication origin. Furthermore, identified publishing and author characteristics are findings of KSSTA analysis and might not represent orthopaedic or knee arthroplasty related research in general. However, various topics were carefully reviewed, and several important trends were identified over a 15-year period.

## Conclusion

Increasing interest in the field of TKA-related surgery has arisen within the last 15 years in KSSTA, accepting an increasing number of articles in each time interval. The main topics showed a significant trend towards the latest techniques at each time interval. While interest in computer-assisted surgery or navigation vanished, interest in optimized perioperative management, rehabilitation and robotic increased. The overall number of references and authors increased which might indicate increased collaboration and globalization over time. Although the absolute number of female first authorships increased, their proportion did not change significantly compared to the first-time interval. There was an increasing number of publications from Asia and North America in KSSTA. The authors hope that the identified publication characteristics might help to better interpret the literature, helps to identify future research topics and serve as a benchmark where knee arthroplasty related research currently stands in KSSTA.

## Abbreviations

KSSTA: Knee Surgery, Sports Traumatology, Arthroscopy
JBJS: Journal of Bone and Joint Surgery
ESKA: ESSKA is the European Society for Sports Traumatology, Knee Surgery and Arthroscopy.
LOE: Level of evidence
PROM: Patient-reported outcome measurement
KSS: Knee Society Score
OKS: Oxford Knee Score
WOMAC: Western Ontario and McMaster Universities Osteoarthritis Index
SF-12: Short Form-12
SF-36: Short Form-36
EQ5d: EuroQol 5d
VAS: Visual Analogue Scale
HSS: Hospital for Special Surgery Score
AKSS: American Knee Society Score
KOOS: Knee Injury and Osteoarthritis Outcome Score
FJS: Forgotten Joint Score
UCLA: University of California at Los Angeles
KSKS & KSFS: Knee Society Knee Score, Knee Society Function Score
PFJ: Patellofemoral Joint Arthroplasty
TKA: Total Knee Arthroplasty
UKA: Unicondylar Knee Arthroplasty
JBJS: The Journal of Bone and Joint Surgery
